# 4-(Methyl­amino)pyridine

**DOI:** 10.1107/S1600536810008986

**Published:** 2010-03-13

**Authors:** Seik Weng Ng

**Affiliations:** a Department of Chemistry, University of Malaya, 50603 Kuala Lumpur, Malaysia

## Abstract

The non-H atoms of the title compound, C_6_H_8_N_2_, lie in a common plane (r.m.s. deviation = 0.034 Å). In the crystal, adjacent mol­ecules are linked by inter­molecular N—H⋯N hydrogen bonds into a zigzag chain running along the *c* axis.

## Related literature

For the non-linear optical activity of co-crystals with substituted 4-nitro­phenol, see; Huang *et al.* (1997 ▶). For the crystal structure of 4-amino­pyridine, see: Anderson *et al.* (2005 ▶) and for that of 4-dimethyl­pyridine, see: Ohms & Guth (1984 ▶).
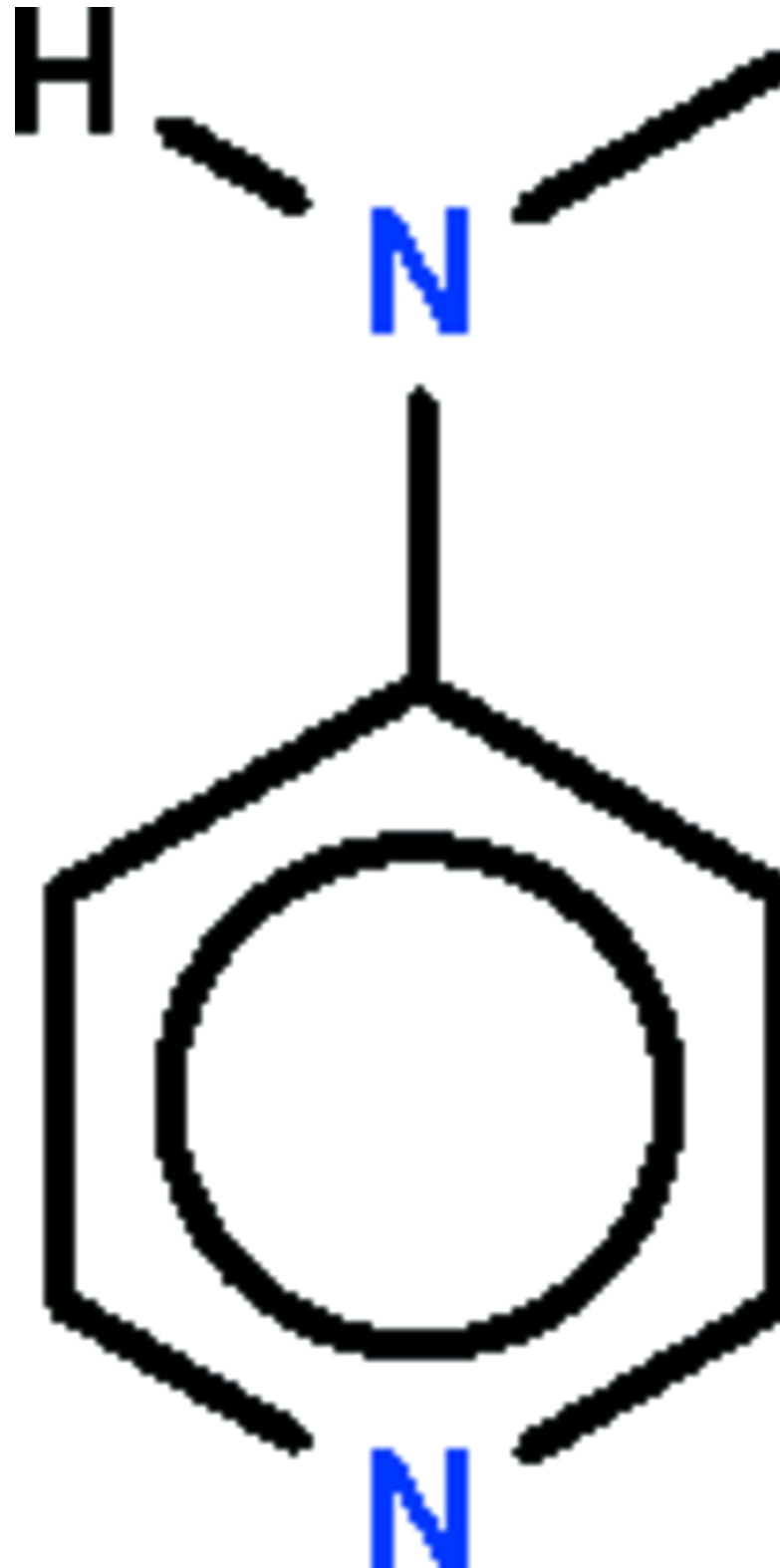


## Experimental

### 

#### Crystal data

C_6_H_8_N_2_
*M* *_r_* = 108.14
Orthorhombic, 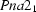

*a* = 6.5645 (18) Å
*b* = 7.1230 (19) Å
*c* = 12.489 (4) Å
*V* = 584.0 (3) Å^3^
*Z* = 4
Mo *K*α radiation
μ = 0.08 mm^−1^
*T* = 100 K
0.12 × 0.12 × 0.02 mm

#### Data collection

Bruker SMART APEX diffractometer
5127 measured reflections
707 independent reflections
521 reflections with *I* > 2σ(*I*)
*R*_int_ = 0.093

#### Refinement

*R*[*F* ^2^ > 2σ(*F* ^2^)] = 0.044
*wR*(*F* ^2^) = 0.102
*S* = 0.99
707 reflections
78 parameters
2 restraints
H atoms treated by a mixture of independent and constrained refinement
Δρ_max_ = 0.18 e Å^−3^
Δρ_min_ = −0.20 e Å^−3^

### 

Data collection: *APEX2* (Bruker, 2009 ▶); cell refinement: *SAINT* (Bruker, 2009 ▶); data reduction: *SAINT*; program(s) used to solve structure: *SHELXS97* (Sheldrick, 2008 ▶); program(s) used to refine structure: *SHELXL97* (Sheldrick, 2008 ▶); molecular graphics: *X-SEED* (Barbour, 2001 ▶); software used to prepare material for publication: *publCIF* (Westrip, 2010 ▶).

## Supplementary Material

Crystal structure: contains datablocks global, I. DOI: 10.1107/S1600536810008986/bt5213sup1.cif

Structure factors: contains datablocks I. DOI: 10.1107/S1600536810008986/bt5213Isup2.hkl

Additional supplementary materials:  crystallographic information; 3D view; checkCIF report

## Figures and Tables

**Table 1:** Hydrogen-bond geometry (Å, °)

*D*—H⋯*A*	*D*—H	H⋯*A*	*D*⋯*A*	*D*—H⋯*A*
N1—H1⋯N2^i^	0.88 (1)	2.06 (1)	2.930 (3)	168 (3)

Symmetry code: (i) 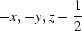.

**Fig. 1.**
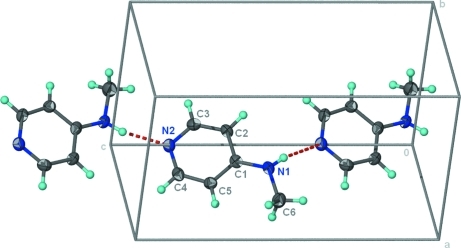
Anisotropic displacement ellipsoid plot (Barbour, 2001) of a portion of the hydrogen-bonded chain structure of 4-methylaminopyridine at the 70% probability level; hydrogen atoms are drawn as spheres of arbitrary radius.

## supplementary crystallographic information

### Comment

The amino nitrogen atom in 4-aminopyridine, a drug used for treating multiple sclerosis, is pyramidal; the amino group engages in a N–H···N hydrogen bonding interaction with adjacent pyridyl rings to generate a chain. The amino group uses its other nitrogen atom to form an N–H···*π*interaction with other pyridyl rings (Anderson *et al.*, 2005).

In the title monomethyl-substituted analogue (Scheme I, Fig. 1), all non-hydrogen atoms lie in a common plane. However, the amino nitrogen atom is slightly pyramidal, this being displaced out of the trigonal plane by 0.18 (2) Å (Σangles 353 °). On the other hand, the amino nitrogen atom in 4-dimethylaminopyridine has unambiguously planar configuration (Ohms & Guth, 1984). In the present structure, adjacent molecules are linked by an N–H···N hydrogen bond to generate a helical chain motif (Table 1).

The compound belongs to a non-centrosymmetric space group, a feature that may render it useful for second-harmonic generation, particularly as it co-crystal with 2-methoxy-4-nitrophenol shows NLO activity (Huang *et al.*, 1997).

### Experimental

4-Methylaminopyridine, as purchased from the Aldrich Chemical Company, is a crystalline material.

### Refinement

Due to the absence of anomalous scatterers, 644 Friedel pairs were merged. Carbon-bound H-atoms were placed in calculated positions (C—H 0.95 Å) and were included in the refinement in the riding model approximation, with *U*(H) set to 1.2*U*_eq_(C). The amino H-atom was located in a difference Fourier map and it was refined with a distance restraint of N–H 0.88±0.01 Å.

### Crystal data

**Table:**

C_6_H_8_N_2_	*F*(000) = 232
*M**_r_* = 108.14	*D*_x_ = 1.230 Mg m^−^^3^
Orthorhombic, *P**n**a*2_1_	Mo *K*α radiation, λ = 0.71073 Å
Hall symbol: P 2c -2n	Cell parameters from 379 reflections
*a* = 6.5645 (18) Å	θ = 3.3–21.5°
*b* = 7.1230 (19) Å	µ = 0.08 mm^−^^1^
*c* = 12.489 (4) Å	*T* = 100 K
*V* = 584.0 (3) Å^3^	Plate, colorless
*Z* = 4	0.12 × 0.12 × 0.02 mm

### Data collection

**Table:**

Bruker SMART APEX diffractometer	521 reflections with *I* > 2σ(*I*)
Radiation source: fine-focus sealed tube	*R*_int_ = 0.093
graphite	θ_max_ = 27.5°, θ_min_ = 3.3°
ω scans	*h* = −7→8
5127 measured reflections	*k* = −9→8
707 independent reflections	*l* = −16→16

### Refinement

**Table:**

Refinement on *F*^2^	Primary atom site location: structure-invariant direct methods
Least-squares matrix: full	Secondary atom site location: difference Fourier map
*R*[*F*^2^ > 2σ(*F*^2^)] = 0.044	Hydrogen site location: inferred from neighbouring sites
*wR*(*F*^2^) = 0.102	H atoms treated by a mixture of independent and constrained refinement
*S* = 0.99	*w* = 1/[σ^2^(*F*_o_^2^) + (0.0518*P*)^2^] where *P* = (*F*_o_^2^ + 2*F*_c_^2^)/3
707 reflections	(Δ/σ)_max_ = 0.001
78 parameters	Δρ_max_ = 0.18 e Å^−^^3^
2 restraints	Δρ_min_ = −0.20 e Å^−^^3^

### Fractional atomic coordinates and isotropic or equivalent isotropic displacement parameters (Å^2^)

**Table:**

	*x*	*y*	*z*	*U*_iso_*/*U*_eq_	
N1	0.2579 (4)	0.0086 (3)	0.49959 (18)	0.0219 (6)	
H1	0.177 (4)	0.027 (5)	0.4440 (19)	0.036 (11)*	
N2	−0.0105 (4)	−0.0122 (3)	0.8044 (2)	0.0232 (6)	
C1	0.1745 (4)	−0.0004 (4)	0.5993 (2)	0.0193 (6)	
C2	−0.0127 (4)	0.0889 (4)	0.6198 (2)	0.0217 (7)	
H2	−0.0813	0.1550	0.5645	0.026*	
C3	−0.0957 (5)	0.0794 (4)	0.7215 (2)	0.0256 (7)	
H3	−0.2215	0.1417	0.7336	0.031*	
C4	0.1690 (4)	−0.0944 (4)	0.7840 (2)	0.0220 (7)	
H4	0.2337	−0.1593	0.8411	0.026*	
C5	0.2673 (4)	−0.0917 (4)	0.6858 (2)	0.0207 (7)	
H5	0.3959	−0.1511	0.6773	0.025*	
C6	0.4412 (4)	−0.0966 (5)	0.4736 (2)	0.0302 (8)	
H6A	0.5534	−0.0546	0.5195	0.045*	
H6B	0.4772	−0.0753	0.3984	0.045*	
H6C	0.4168	−0.2307	0.4853	0.045*	

### Atomic displacement parameters (Å^2^)

**Table:**

	*U*^11^	*U*^22^	*U*^33^	*U*^12^	*U*^13^	*U*^23^
N1	0.0237 (13)	0.0242 (14)	0.0178 (12)	0.0021 (12)	−0.0023 (10)	0.0025 (12)
N2	0.0277 (14)	0.0232 (12)	0.0189 (11)	0.0006 (12)	0.0005 (11)	−0.0001 (13)
C1	0.0189 (15)	0.0192 (15)	0.0198 (14)	−0.0048 (13)	−0.0055 (12)	−0.0010 (12)
C2	0.0231 (17)	0.0218 (16)	0.0201 (13)	0.0003 (13)	−0.0031 (12)	0.0033 (13)
C3	0.0293 (18)	0.0210 (16)	0.0264 (16)	0.0046 (14)	0.0013 (14)	−0.0005 (14)
C4	0.0248 (16)	0.0223 (16)	0.0188 (14)	−0.0016 (13)	−0.0039 (12)	0.0009 (13)
C5	0.0196 (16)	0.0205 (16)	0.0219 (14)	0.0009 (13)	−0.0024 (12)	−0.0011 (14)
C6	0.0242 (16)	0.0408 (19)	0.0256 (16)	0.0051 (14)	0.0028 (14)	0.0005 (14)

### Geometric parameters (Å, °)

**Table:**

N1—C1	1.362 (4)	C2—H2	0.9500
N1—C6	1.454 (4)	C3—H3	0.9500
N1—H1	0.88 (1)	C4—C5	1.386 (4)
N2—C4	1.340 (4)	C4—H4	0.9500
N2—C3	1.345 (4)	C5—H5	0.9500
C1—C5	1.401 (4)	C6—H6A	0.9800
C1—C2	1.407 (4)	C6—H6B	0.9800
C2—C3	1.384 (4)	C6—H6C	0.9800
			
C1—N1—C6	120.8 (2)	N2—C4—C5	124.8 (3)
C1—N1—H1	119 (2)	N2—C4—H4	117.6
C6—N1—H1	113 (2)	C5—C4—H4	117.6
C4—N2—C3	115.6 (3)	C4—C5—C1	119.1 (3)
N1—C1—C5	123.5 (3)	C4—C5—H5	120.4
N1—C1—C2	119.8 (3)	C1—C5—H5	120.4
C5—C1—C2	116.7 (3)	N1—C6—H6A	109.5
C3—C2—C1	119.3 (3)	N1—C6—H6B	109.5
C3—C2—H2	120.4	H6A—C6—H6B	109.5
C1—C2—H2	120.4	N1—C6—H6C	109.5
N2—C3—C2	124.5 (3)	H6A—C6—H6C	109.5
N2—C3—H3	117.7	H6B—C6—H6C	109.5
C2—C3—H3	117.7		
			
C6—N1—C1—C5	7.2 (4)	C1—C2—C3—N2	−0.6 (5)
C6—N1—C1—C2	−174.3 (3)	C3—N2—C4—C5	−0.5 (5)
N1—C1—C2—C3	−179.8 (3)	N2—C4—C5—C1	−1.2 (5)
C5—C1—C2—C3	−1.1 (4)	N1—C1—C5—C4	−179.4 (3)
C4—N2—C3—C2	1.5 (5)	C2—C1—C5—C4	2.0 (4)

### Hydrogen-bond geometry (Å, °)

**Table:**

*D*—H···*A*	*D*—H	H···*A*	*D*···*A*	*D*—H···*A*
N1—H1···N2^i^	0.88 (1)	2.06 (1)	2.930 (3)	168 (3)

Symmetry codes: (i) −*x*, −*y*, *z*−1/2.
